# Supplementary material for: Active Brownian particles and run-and-tumble particles separate inside a maze
Source: Sci Rep. 2016 Nov 23;6:37670. doi: 10.1038/srep37670 (PMC5120314; doi:10.1038/srep37670)
Supplement: Supplementary Information [file srep37670-s1.pdf]

Supplementary Material: Active brownian particles and run-and-tumble particles separate inside a maze

Maryam Khatami,[1,2] Katrin Wolff,[2] Oliver Pohl,[2] M. Reza Ejtehadi,[1,3] and Holger Stark[2]

[1]*Department of Physics, Sharif University of Technology, P.O. Box 11155-9161, Tehran, Iran*
[2]*Institut für Theoretische Physik, Technische Universität Berlin, Hardenbergstrasse 36, 10623 Berlin, Germany*
[3]*School of Nano-Science, Institute for Research in Fundamental Sciences (IPM), P. O. Box 19395-5531, Tehran, Iran*

MOVIES

All of the movies are particle-based simulations of the model discussed in the main text. Red disk is a symbol for the particle with an attached black arrow showing its self-propulsion velocity direction.

- Supplementary movie 1: ABP with $P_r = 15$ in the outwards case of the circular maze.
- Supplementary movie 2: RTP with $P_r = 15$ in the outwards case of the circular maze.
- Supplementary movie 3: ABP with $P_r = 15$ in the inwards case of the circular maze.
- Supplementary movie 4: RTP with $P_r = 15$ in the inwards case of the circular maze.
- Supplementary movie 5: RTP with $P_r = 15$ in the inwards case of the square maze.
- Supplementary movie 6: ABP with $P_r = 15$ in the inwards case of the square maze.

BROWNIAN DYNAMICS SIMULATION

Since the main results of this work are obtained from Brownian dynamics simulations, the accuracy of the numerical method is important. We rescale all lengths and times by R and $30R/v_0$, resepectively, and vary the particle persistence time τ_r between 0.1 and 5. So, it roughly is of the order of one. With this knowledge we choose the simulation time step dt equal to $dt = 10^{-4}$ such that dt/τ_r is in the range $[2 \times 10^{-5}, 10^{-3}]$. Furthermore, in our reduced units particle speed is $v_0 = 30$. With this set of parameters, we are sure that in each time step, the particle displacement is at least 10 times smaller than the maximum gap size, $0.05R$, between particles and channel walls, so that we clearly resolve the lateral motion of the particles in the channels. Note that the temporal evolution of particle position is coupled to the evolution of its orientation, which obeys the Langevin equation (3) in the main text. Therefore, by solving the position equation with an Euler scheme and the orientation equation with the Ito approach, the overall numerical error is of order $O(\sqrt{\Delta t})$.

MEAN FIRST-PASSAGE TIME

Consider a particle undergoing a pure rotational diffusion in two dimensions. The probability distribution of the particle to have orientation θ at time t obeys the one-dimensional diffusion equation,

$$\frac{\partial}{\partial t} P(\theta, t) = D_r \frac{\partial^2}{\partial \theta^2} P(\theta, t). \tag{1}$$

Let the initial orientation of the particle be θ_0 between two absorbing boundaries at θ_1 and θ_2, which means $P(\theta_1, t) = P(\theta_2, t) = 0$. One can write the backward diffusion equation for the conditional probability of the system[1, 2],

$$\frac{\partial}{\partial t} p(\theta, t|\theta_0, 0) = D_r \frac{\partial^2}{\partial \theta_0^2} p(\theta, t|\theta_0, 0). \tag{2}$$

The survival probability that the particle orientation is still between θ_1 and θ_2 at time t is then derived from[3]

$$S(\theta_0, t) = \int_{\theta_1}^{\theta_2} d\theta p(\theta, t|\theta_0, 0). \tag{3}$$

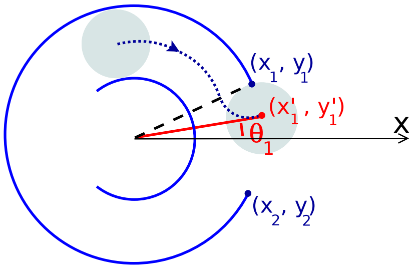

FIG. 1. **Reduced opening angle.** The particle is shown with gray disk. The dashed black line connects the center of maze to the point (x_1, y_1) and determines the angle of the opening. By looking at the dotted curve, which is a sample trajectory for the center of the particle, it becomes clear that the reduced opening angle θ_1 for calculating the mean absorption time has to take into account the finite particle radius. The reduced opening coordinates are (x_1', y_1'). We use θ_1 to evaluate $T(\theta_0)$ from Eq. (6).

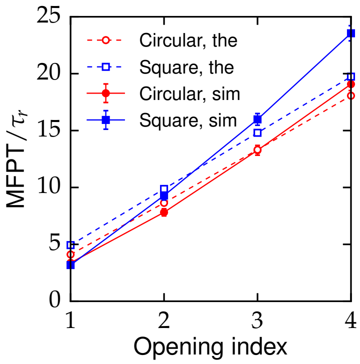

FIG. 2. **Comparison between simulation results (sim) for $P_r = 75$ and theoretical results (the) calculated from Eq. (6) of the main text for the circular and square mazes.**

The mean time to be absorbed at either boundary can be calculated from the survival probability with (see [2] for more details)

$$T(\theta_0) = \int_0^\infty dt S(\theta_0, t). \tag{4}$$

By using equations (3) and (4) in equation (2), a second order differential equation is derived for $T(\theta_0)$,

$$-1 = D_r \frac{\partial^2}{\partial \theta_0^2} T(\theta_0). \tag{5}$$

This equation can be solved by considering $T(\theta_1) = T(\theta_2) = 0$ as boundary conditions, which gives

$$T(\theta_0) = \frac{(\theta_0 - \theta_1)(\theta_2 - \theta_0)}{2D_r}. \tag{6}$$

opening index	1	2	3	4
shell radius (r)	1.80	3.85	5.90	7.95
$2\theta_1$	0.55	0.26	0.17	0.12

TABLE I. **Opening angles in units of radians for different shells of the circular maze.**

As shown in Fig. 3(a) in the main text, for the circular maze the escape angles θ_1 and θ_2 (or reduced opening angles) are related by $\theta_2 = 2\pi - \theta_1$ to each other. They are also closely linked to the coordinates (x_1, y_1) of both sides

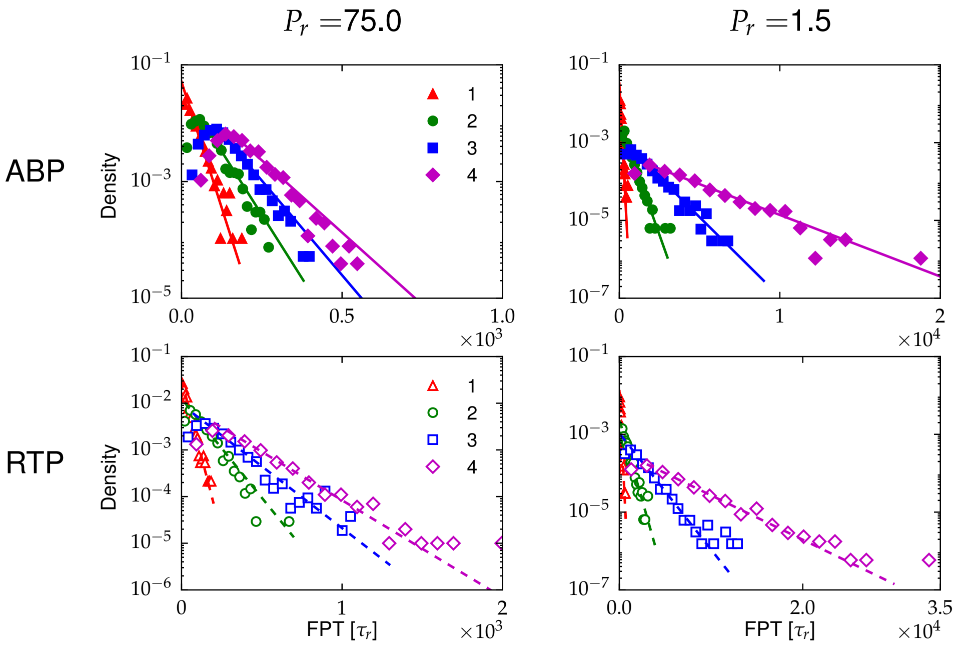

FIG. 3. Probability distribution of FPTs for ABP (first row) and RTP (second row) inside the circular maze in the outwards case. The left column corresponds to $P_r = 75$ and the right column corresponds to $P_r = 1.5$. Lines indicate an exponential fit to 1000 realizations in each case.

of the opening, as illustrated in Fig. 1, taking into account the non-zero size of the active particles. In concrete, the coordinates (x_1, y_1) can be calculated from the width $3R$ of each opening. The reduced opening angle follows from the geometrical relation $\theta_1 = \cos^{-1}(x_1'/r)$ [see Fig. 1], where (x_1', y_1') correspond to the reduced opening coordinates. They are obtained by simultaneously solving the two following equations

$$(x_1 - x_1')^2 + (y_1 - y_1')^2 = R, \tag{7}$$

$$x_1^2 + y_1^2 = r^2. \tag{8}$$

Results for θ_1 are summarized in Table I. Note that this choice of angles overestimates the MFPT since especially at large P_r the particle is able to escape from the opening also with larger orientation angles, which have not fully relaxed to the angle belonging to (x_1', y_1'). A quantitative comparison between the theoretical values calculated from Eq. (6) and numerical results for the largest P_r are shown in Fig. 2 for both the circular and square mazes.

PROBABILITY DISTRIBUTIONS OF FIRST-PASSAGE TIMES

We have looked at the distribution of first-passage times (FPTs) for the circular maze. Both in the outwards (Fig. 3) and inwards case (Fig. 4) the distributions decay exponentially for both types of particles.

RATE THEORY

Consider a particle hopping between adjacent sites of a one-dimensional lattice with five sites. The time evolution of the probability for being at each site is governed by a linear rate equation

$$\frac{d}{dt}|\psi(t)\rangle = -\mathbf{Q}|\psi(t)\rangle, \tag{9}$$

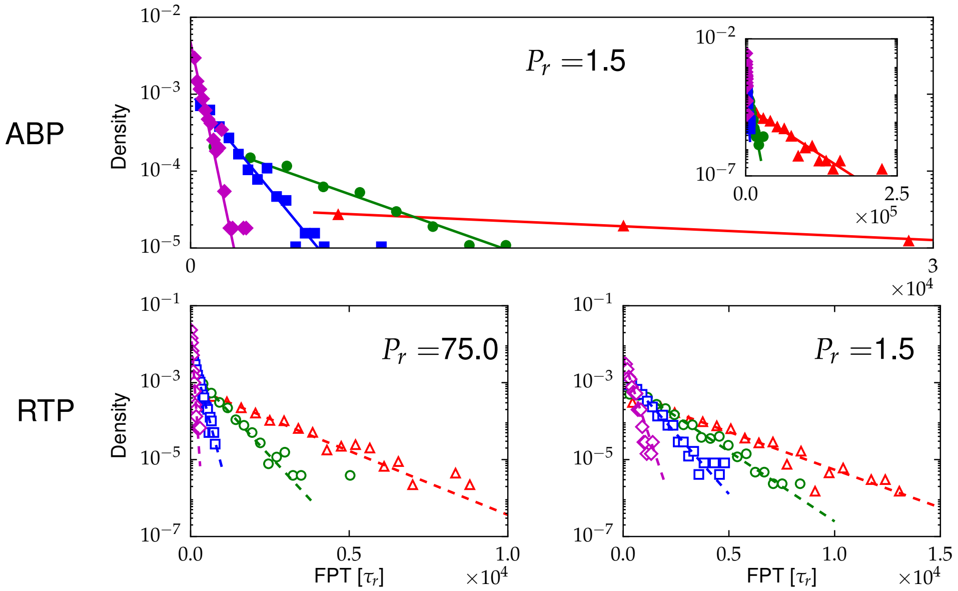

FIG. 4. Probability distribution of FPTs for ABP (first row) and RTP (second row) inside the circular maze for the inwards case. In (a) the inset shows a wider range of data. Due to very long simulation times and limitations in computational resources, extracting the distribution for ABP with $P_r = 75$ was not possible. Lines indicate an exponential fit to 500 (first row) and 1000 (second row) realizations for each case.

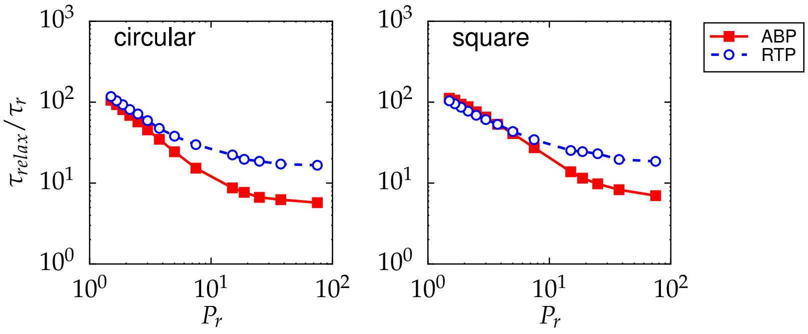

FIG. 5. **Relaxation time for ABP and RTP in the circular (a) and square (b) mazes.** Symbols are extracted from rate theory analysis and connected by lines to guide the eyes.

where $|\psi(t)\rangle = |p_1(t), p_2(t), p_3(t), p_4(t), p_5(t)\rangle$ is the state vector of the system and $p_i(t)$ the probability that the particle is found at site i $(i = 1, 2, 3, 4, 5)$ at time t. $\mathbf{Q}$ is the transition rate matrix that can be written as

$$\mathbf{Q} = \begin{pmatrix} k_{12} & -k_{21} & 0 & 0 & 0 \\ -k_{12} & k_{21}+k_{23} & -k_{32} & 0 & 0 \\ 0 & -k_{23} & k_{32}+k_{34} & -k_{43} & 0 \\ 0 & 0 & -k_{34} & k_{43}+k_{45} & -k_{54} \\ 0 & 0 & 0 & -k_{45} & k_{54} \end{pmatrix} \tag{10}$$

where k_{ij} is the transition rate to jump from site i to site j. Practically, we measure the mean time that a particle stays at each of the sites before it hops to a neighboring site and also the frequency of these transitions. With these two, we can calculate all the transition rates between adjacent sites.

In general, the transition matrix $\mathbf{Q}$ has one zero eigenvalue, where the corresponding eigenstate corresponds to the stationary state of the system $(dp_i/dt = 0)$. Other eigenvalues are positive and show the transient behavior. So, the relaxation time for the system to reach its stationary state is calculated as $\tau_{\text{relax}} = 1/\lambda_r$, where λ_r is the smallest non-zero eigenvalue of matrix $\mathbf{Q}$.

The relaxation time was determined for both of the nested mazes (Fig. 5). The results show that for large P_r

both particles reach their stationary state quite fast. They also show that the particle in the circular maze reaches its stationary state faster than in the square maze, which is a consequence of curvature.

---

[1] M. Muthukumar, *Polymer Translocation* (CRC Press, 2016) Chap. 6.
[2] C. W. Gardiner, *Handbook of stochastic methods for physics, chemistry and the natural sciences*, 3rd ed., Springer Series in Synergetics, Vol. 13 (Springer-Verlag, Berlin, 2004) Chap. 5.
[3] Sidney Redner, *A guide to first-passage processes* (Cambridge University Press, 2001) Chap. 2.
